# 3D-Printed Rehabilitation and Immediate Loading in a Completely Edentulous Patient With Computed Guided Surgery: A Case Report With a 24-Month Follow-Up

**DOI:** 10.7759/cureus.84285

**Published:** 2025-05-17

**Authors:** João C Vicente de Barros, Fabrício L Gebrin, Jamil A Shibli, Márcio de Carvalho Formiga, Tárcio H Ishimine Skiba

**Affiliations:** 1 Implantology and Oral Rehabilitation, Study, Research and Dental Service (SOEP), Porto Velho, BRA; 2 Periodontology, Guarulhos University, Guarulhos, BRA; 3 Dentistry, Albert Einstein Israelite Faculty of Health Sciences, Albert Einstein Israelite Hospital, São Paulo, BRA; 4 Oral Implantology, Universidade do Vale do Itajaí (UNIVALI) São José, Florianopolis, BRA; 5 Pharmaceutics, Universidade do Vale do Itajaí (UNIVALI), Itajaí, BRA

**Keywords:** 3d printing, digital workflow, guided implant surgery, implant osseointegration, virtual planning

## Abstract

Implant-supported fixed rehabilitations are a reliable solution to restore aesthetics and function to edentulous patients. Currently, practitioners can use digital flow with computer-aided design & computer-aided manufacturing (CAD/CAM) technology to enhance their productivity by reducing working time and providing more comfort to patients. This report presents a clinical case in which the digital flow was used to restore the inferior edentulous arch in a 55-year-old female patient with an implant-based Misch's FP3 denture. Digital technology provides tridimensional planning, surgical computed guides, and printed temporary rehabilitation. This case report portrays the feasibility and ease that the digital flow provides for both the surgeon and the patient.

## Introduction

Prosthetic rehabilitation of patients with total edentulism remains one of the main challenges in dentistry. The practitioner ought to restore function and esthetics most predictably while employing the shortest and most comfortable working time, sometimes without important clinical parameters to aid rehabilitation [1,2]. The implant-supported full arch fixed rehabilitation has become an accepted treatment option for such cases, more specifically in immediate loading cases, thanks to the evolving technology in all the fields of digital dentistry, assuring more predictability and precision in oral rehabilitation [3].

Regarding evolving technology, digital workflow may provide a virtual tridimensional preoperative planning, where we can visualize the final restoration before it starts, in addition to a 3D printing surgical guide and the possibility of printing a temporary prosthesis before the surgical phase [4,5]. The use of this technology may decrease the need for many appointments and, at the same time, ensure more accuracy in the final result. The main advantage of digital planning and the utilization of a surgical guide is the possibility of placing the dental implants in an optimal tridimensional position, which is a key point to a long-term successful case.

Computer-guided, flapless implant surgery offers the advantages of high-precision implant placement, shorter surgical time, less trauma and bleeding, and fewer postoperative complications. It is, however, a highly technique-sensitive procedure at all stages that begins with the virtual planning and finishes with the surgical procedure, all of which need to be accurately performed [6]. Therefore, this report presents a clinical case of a full-arch rehabilitation with screw-retained on five 3D-printed implants placed by computer-guided surgery that was submitted to an immediate loading protocol, and the 24-month follow-up.

## Case presentation

**Figure 1:**
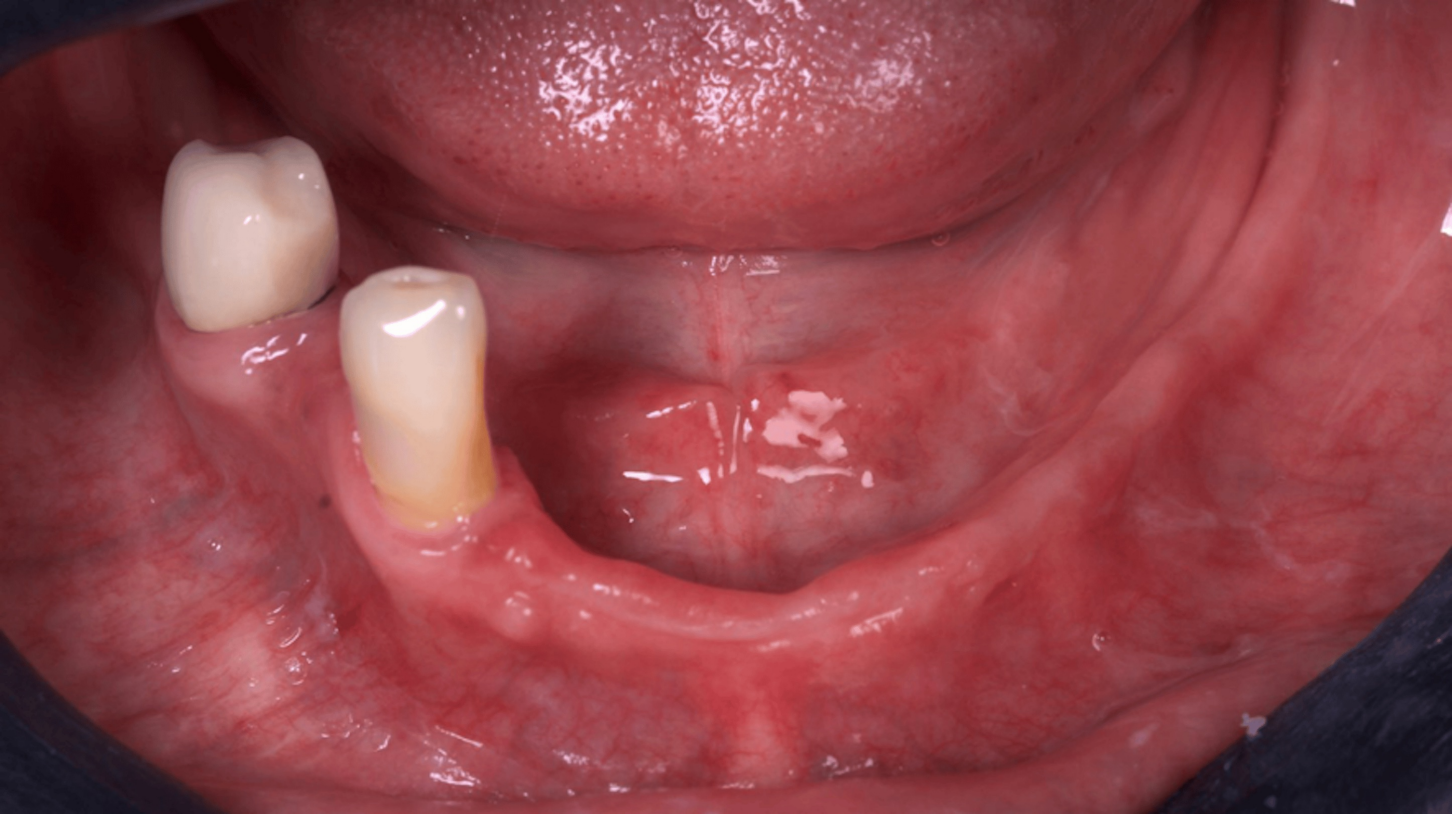
Intraoral view of the patient's mandible

A 55-year-old female was referred to the clinic of the Department of Implantology and Oral Rehabilitation at the Study, Research and Dental Service (SOEP) (Porto Velho, BRA), to treat her lower jaw with a screw-retained full arch rehabilitation over implants. During the intraoral examination, we could observe that she presented only the right first and second premolars (Figure 1). The choice of full-arch rehabilitation with the flapless placement of five 3D-printed implants, followed by immediate loading, was proposed and accepted by the patient, who used to have a removable partial denture, and wanted a fixed alternative for her rehabilitation, with minimal surgeries.

**Figure 2:**
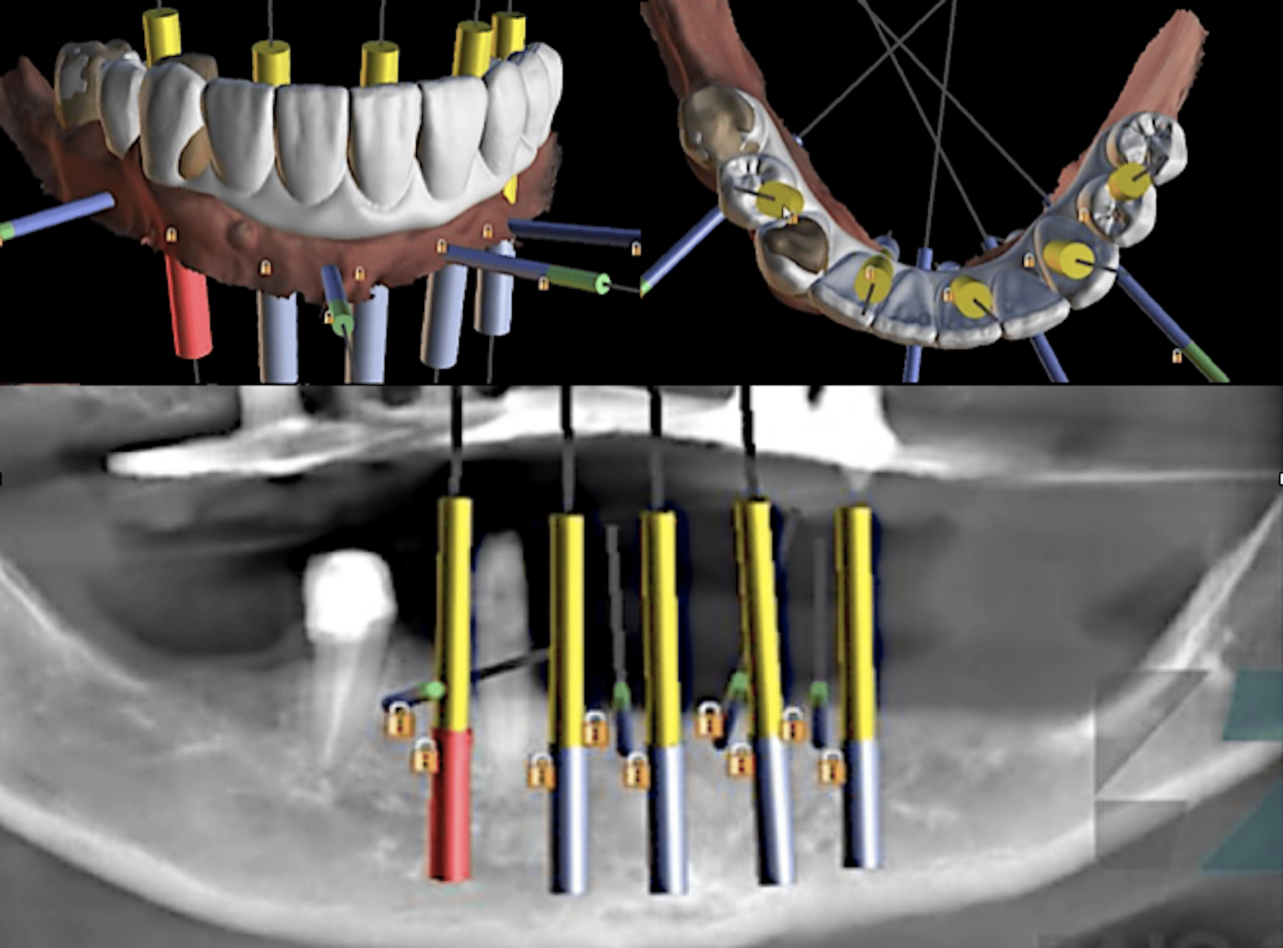
The 3D digital planning of the implants conducted at Raio 3D (Porto Velho, BRA)

**Figure 3:**
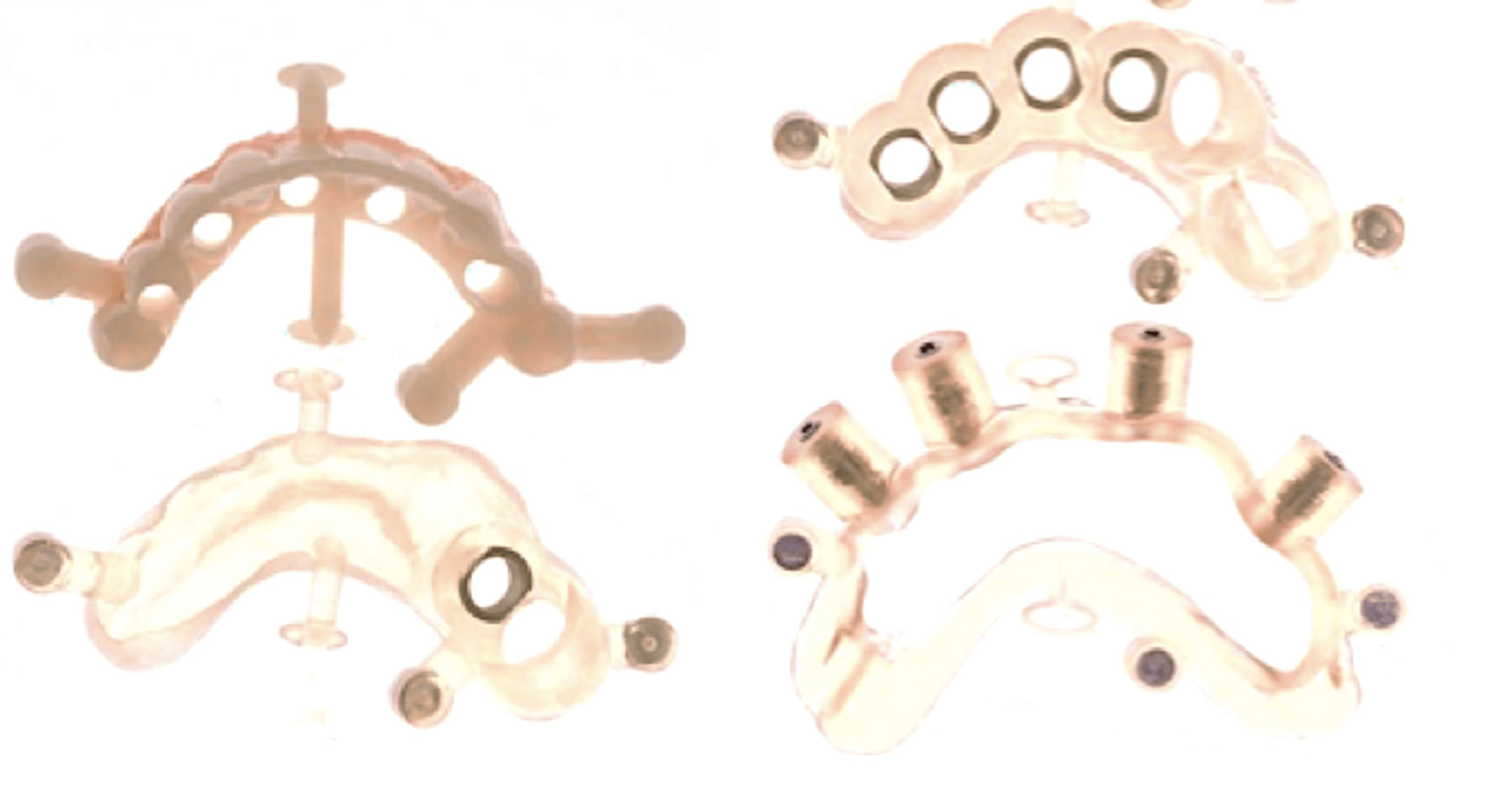
Set of stackable guides utilized for this clinical case

The full digital workflow was utilized in this case to improve predictability and long-term stability and decrease the clinical steps to rehabilitation completion. After intraoral scanning, the case was digitally planned using CT scan and computer software that granted a tridimensional view of the arches and consequent assessment of the ideal tridimensional position of five dental implants placed at the inter-foraminal region (Figure 2). A set of surgical stackable guides was printed to be used intraoperatively, aimed to guide the optimal tridimensional flapless implant placement with immediate loading (Figure 3). Four 3.5 mm x 11.5 mm implants and one 4 mm x 10 mm implant (Plenum Bioengenharia, Jundiaí, SP, BRA), commercially produced by additive manufacturing, using titanium powder grade 23, were planned. The patient approved the treatment plan and provided a written informed consent to publish this report.

The surgery was performed under local anesthesia, with 2% articaine and 1:100.000 epinephrine. The patient was prescribed with 0.12% chlorexidine rinse right before surgical draping. One hour prior to the procedure, 8 mg dexamethasone and 1 gm of amoxicillin were prescribed. Moreover, 500 mg of amoxicillin every eight hours for seven days and ibuprofen 600 mg every six hours for three days postoperatively for pain control were prescribed.

**Figure 4:**
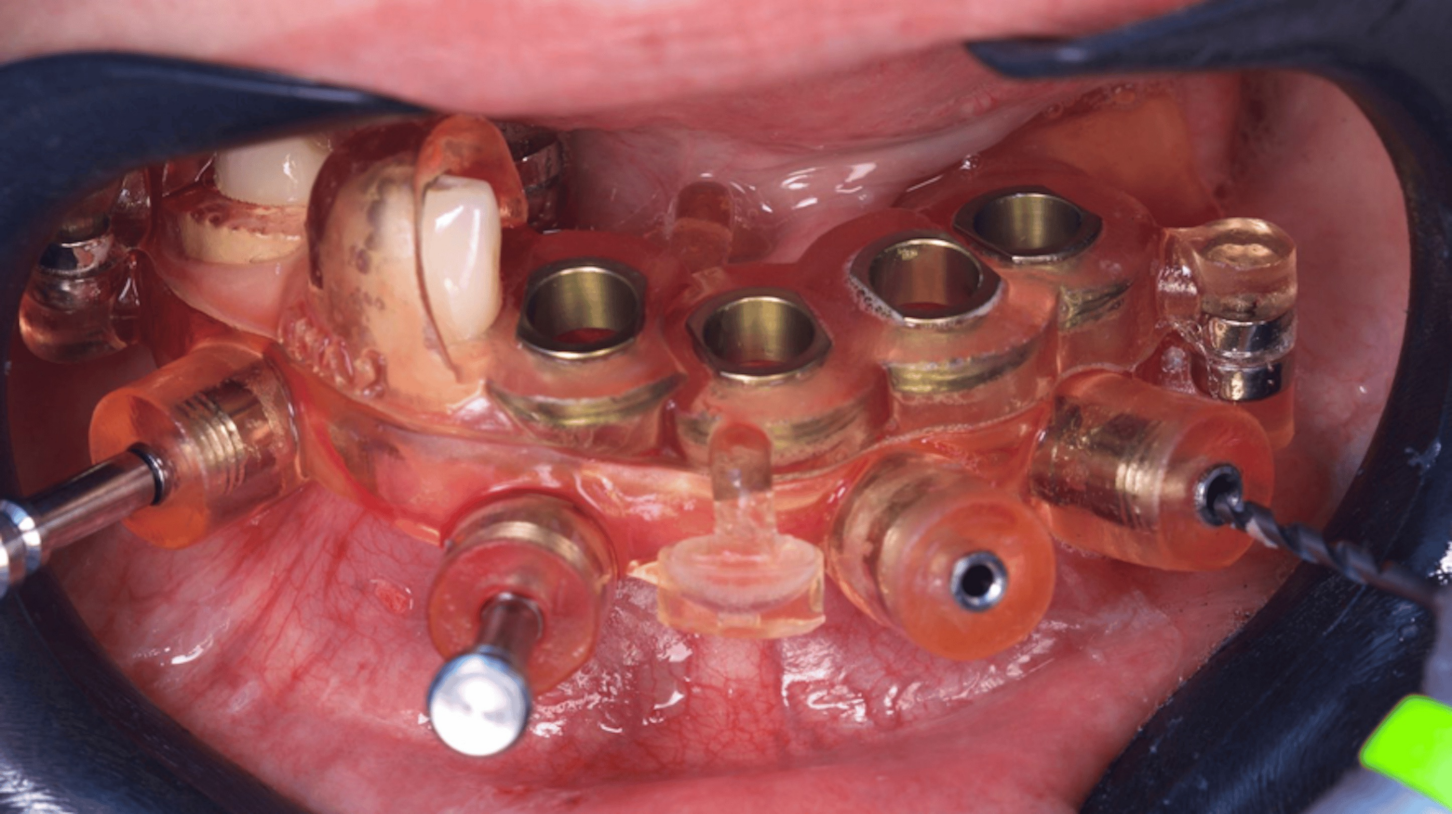
Buccal view of the two stackable guides installed and stabilized with three pins

**Figure 5:**
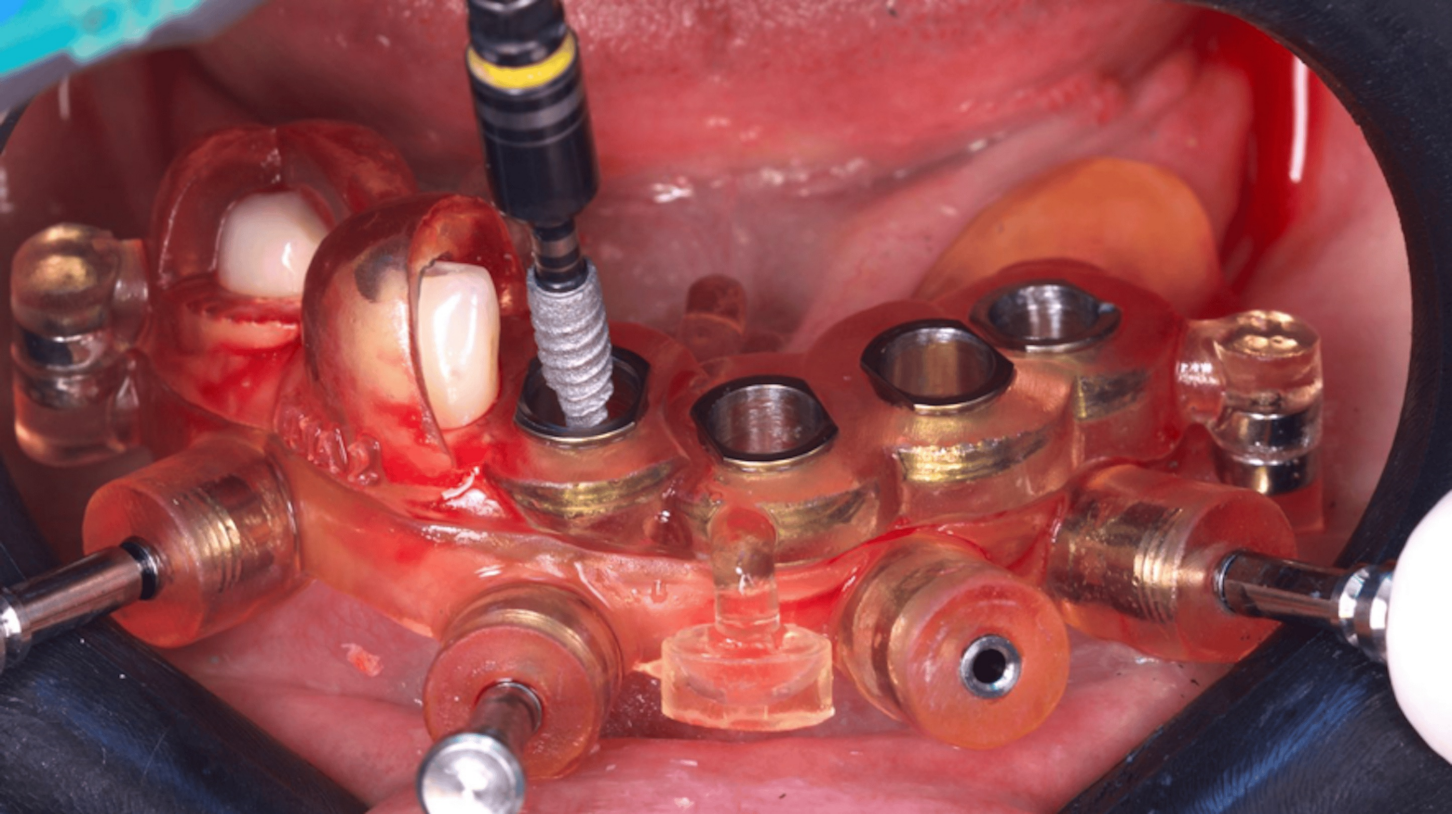
Implant placement through the printed surgical guide

**Figure 6:**
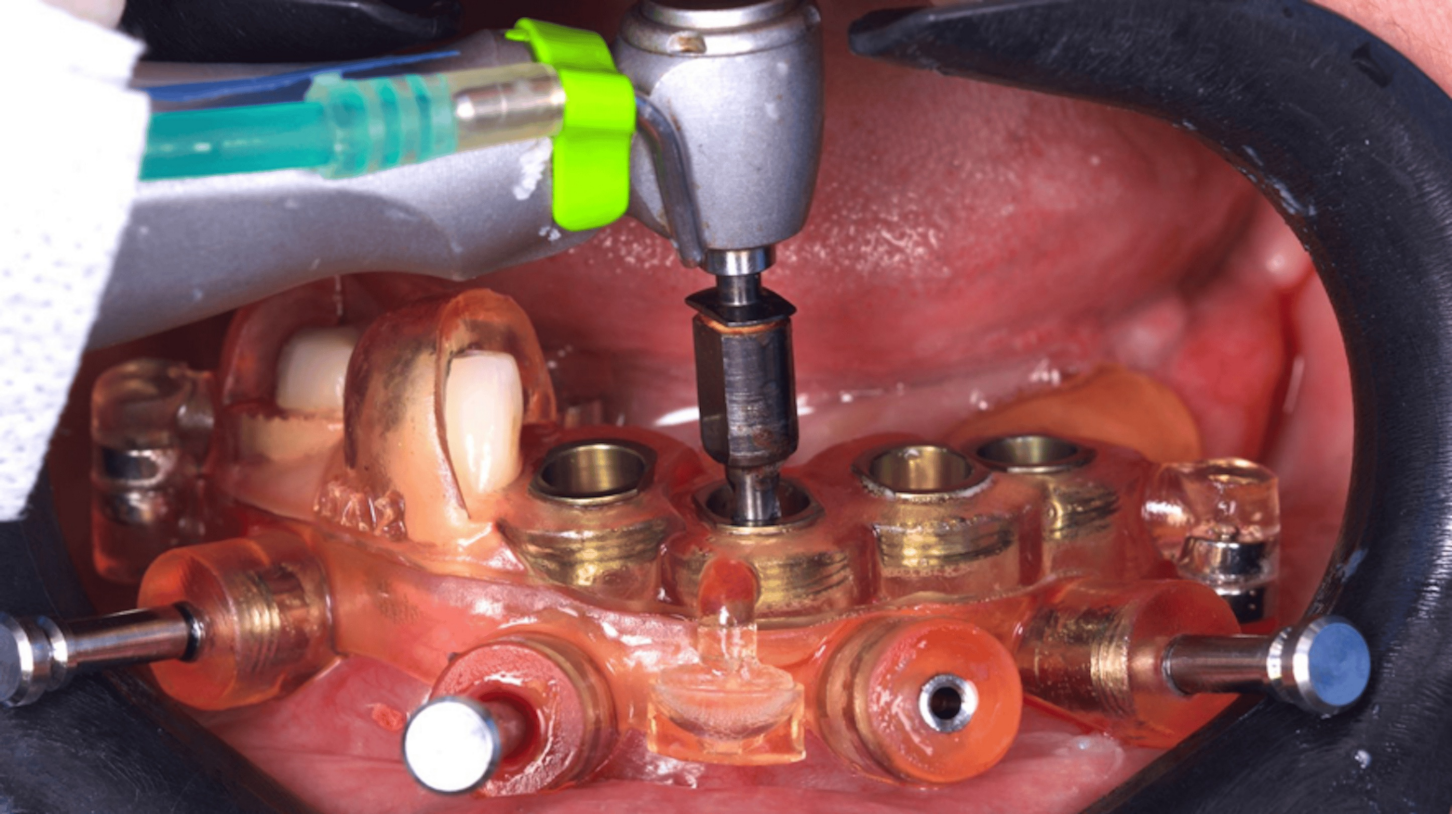
Flapless placement of the second implant

**Figure 7:**
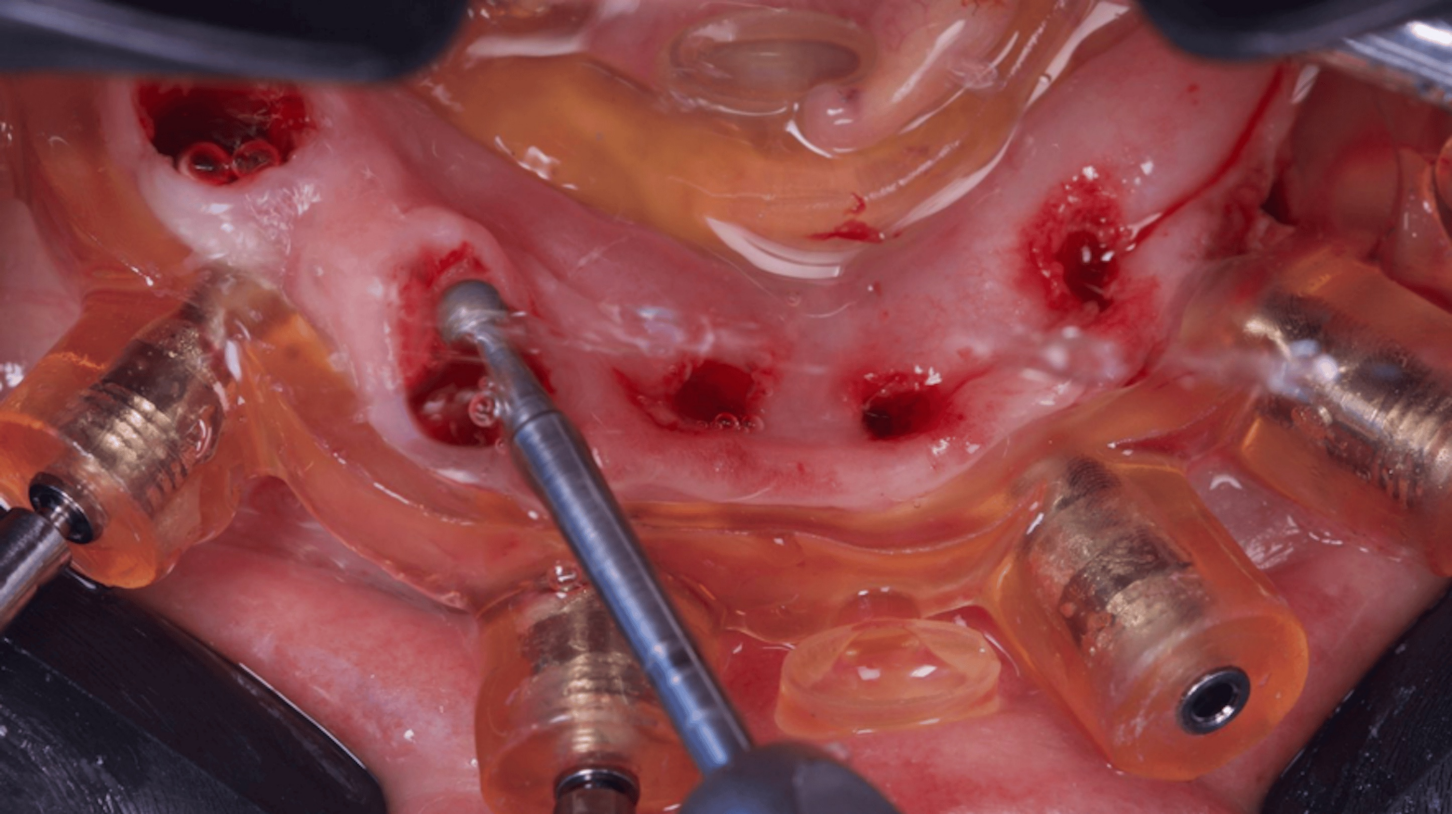
Osteotomy of the alveolus after teeth extraction

**Figure 8:**
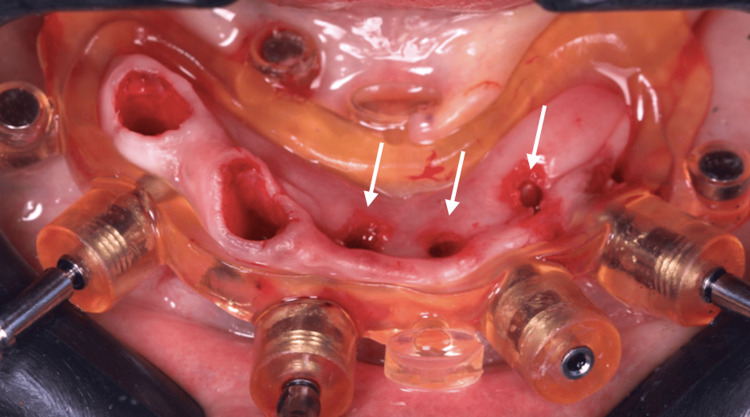
View of the lower jaw mucosa after osteoplasty and the placement of three implants (white arrows)

After anesthesia, two stackable guides were installed in the lower jaw. The first one with 3-pin and the second through magnets on the top of the other, so that three 3.5 mm x 11.5 mm implants could be placed in a flapless approach (Figures 4-6). Subsequently, the magnet-inserted implant guide was removed, and the teeth that aided in the stabilization of the first guide were extracted with minimal trauma, along with the performance of a small osteoplasty in the first premolar alveolus employing a hand-piece spherical bur (Figures 7-8).

**Figure 9:**
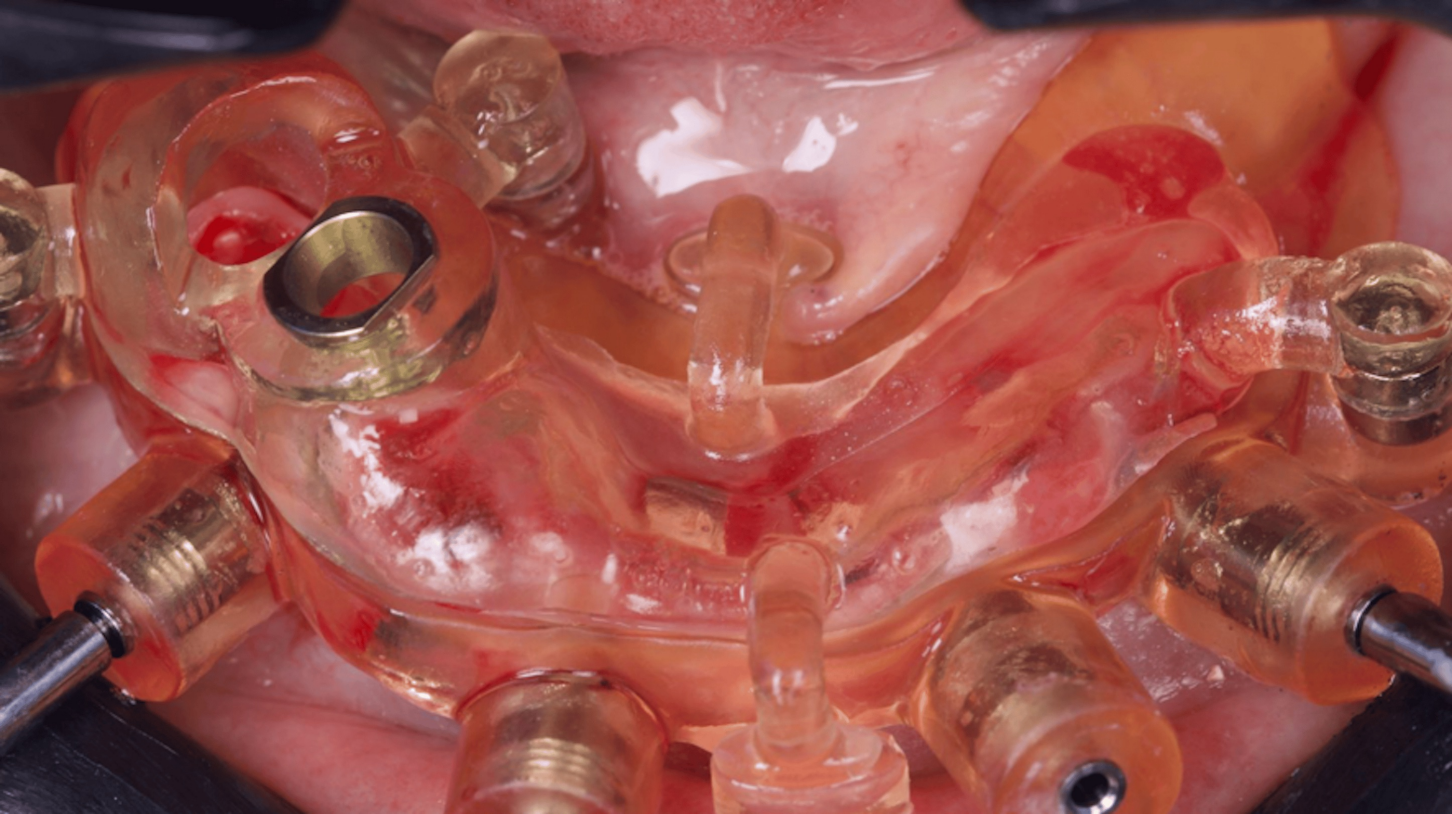
The second guide placed in position by magnets

**Figure 10:**
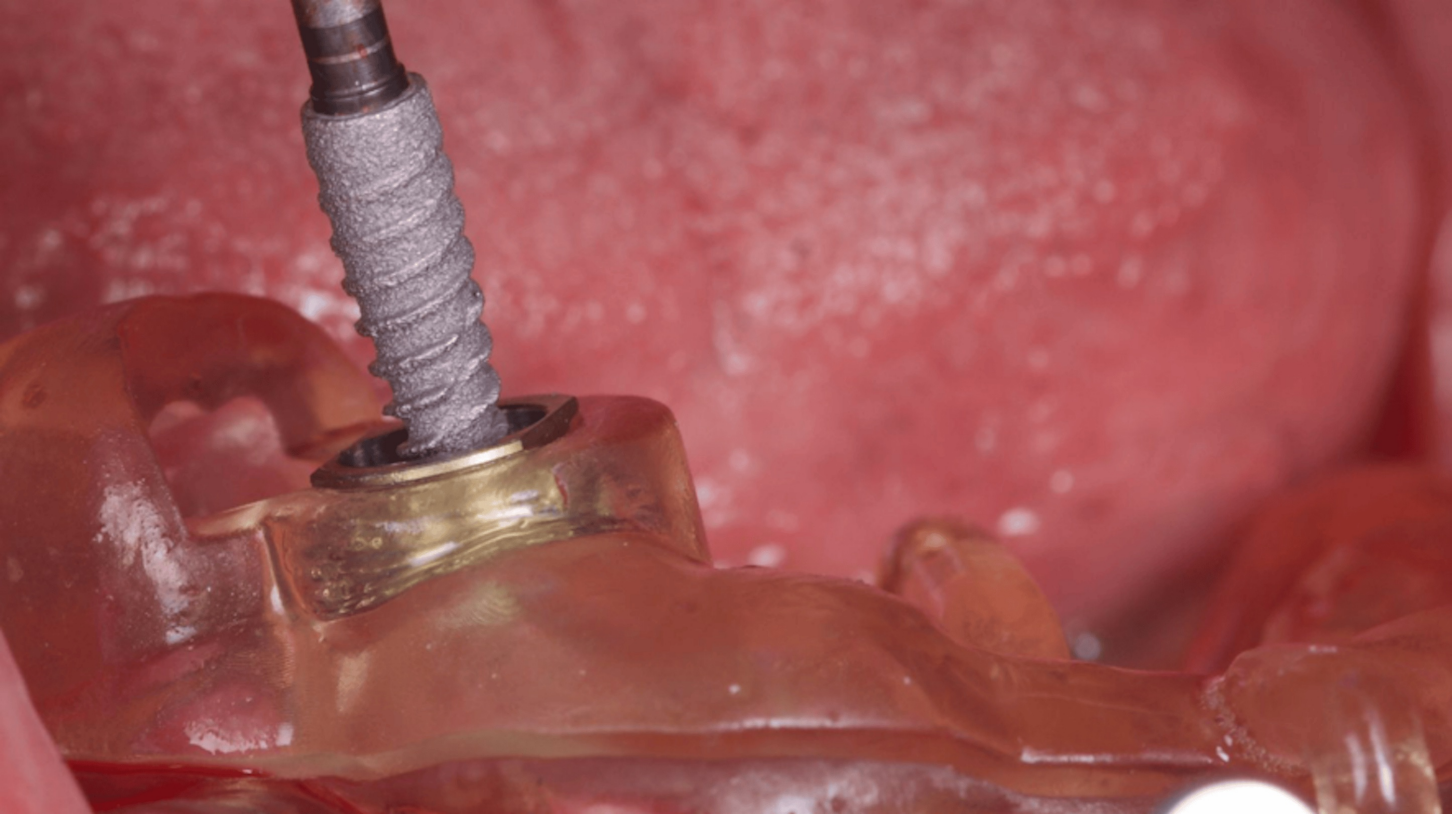
Placement of a 3D implant in the first right premolar alveolus

**Figure 11:**
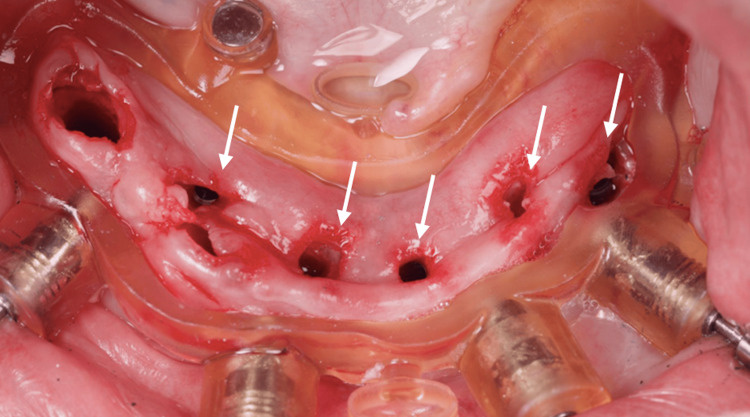
View of the lower jaw mucosa after all implants have been placed (white arrows)

A second guide was placed utilizing magnets to guide the placement of the 4 mm x 10 mm implant in the first right premolar area, whereas a 3.5 x 11.5 mm implant could also be placed in the second right premolar alveolus (Figures 9-11). 

**Figure 12:**
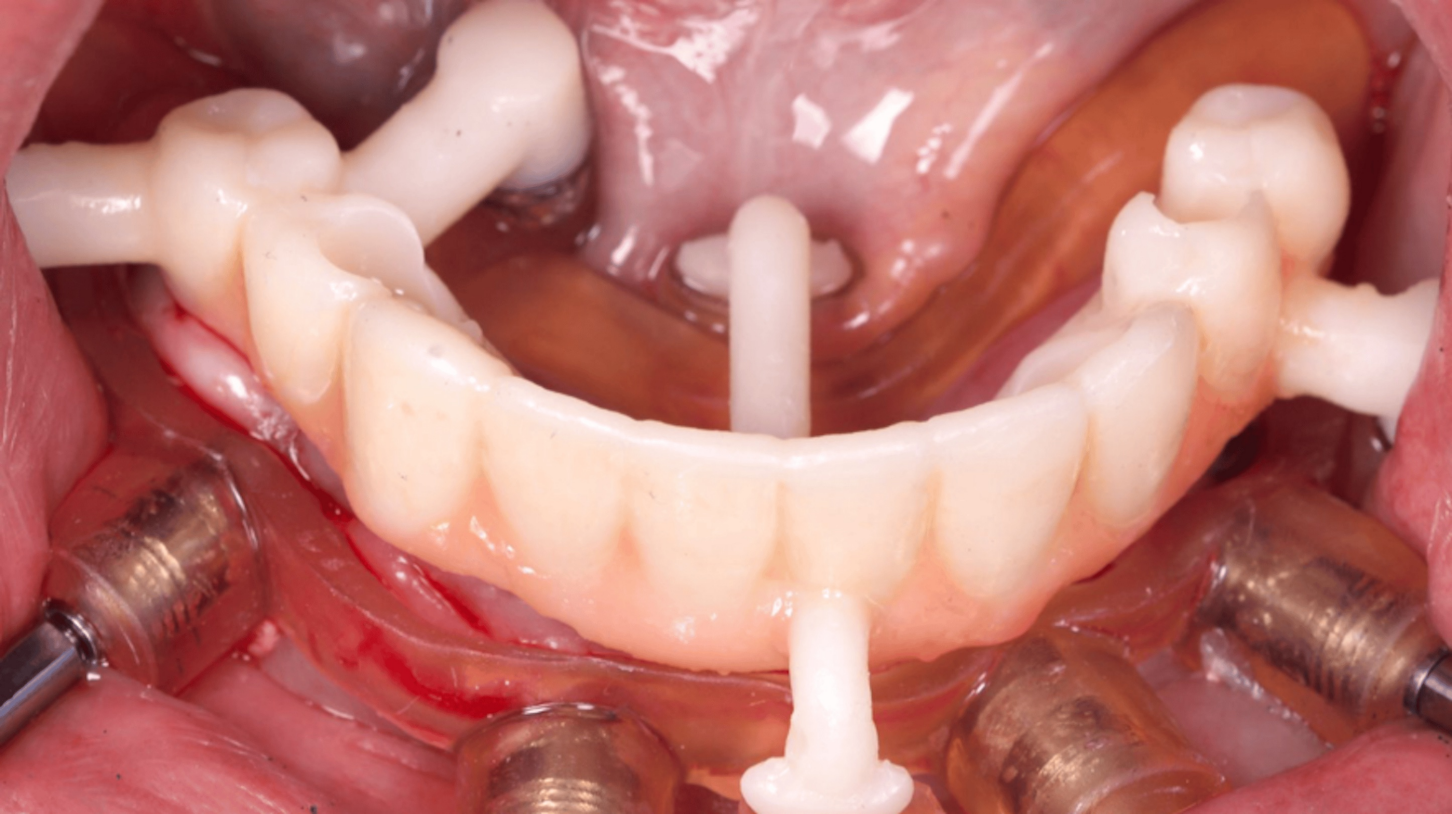
View of the resin-printed temporary prosthesis installed over the surgical guide still in position

**Figure 13:**
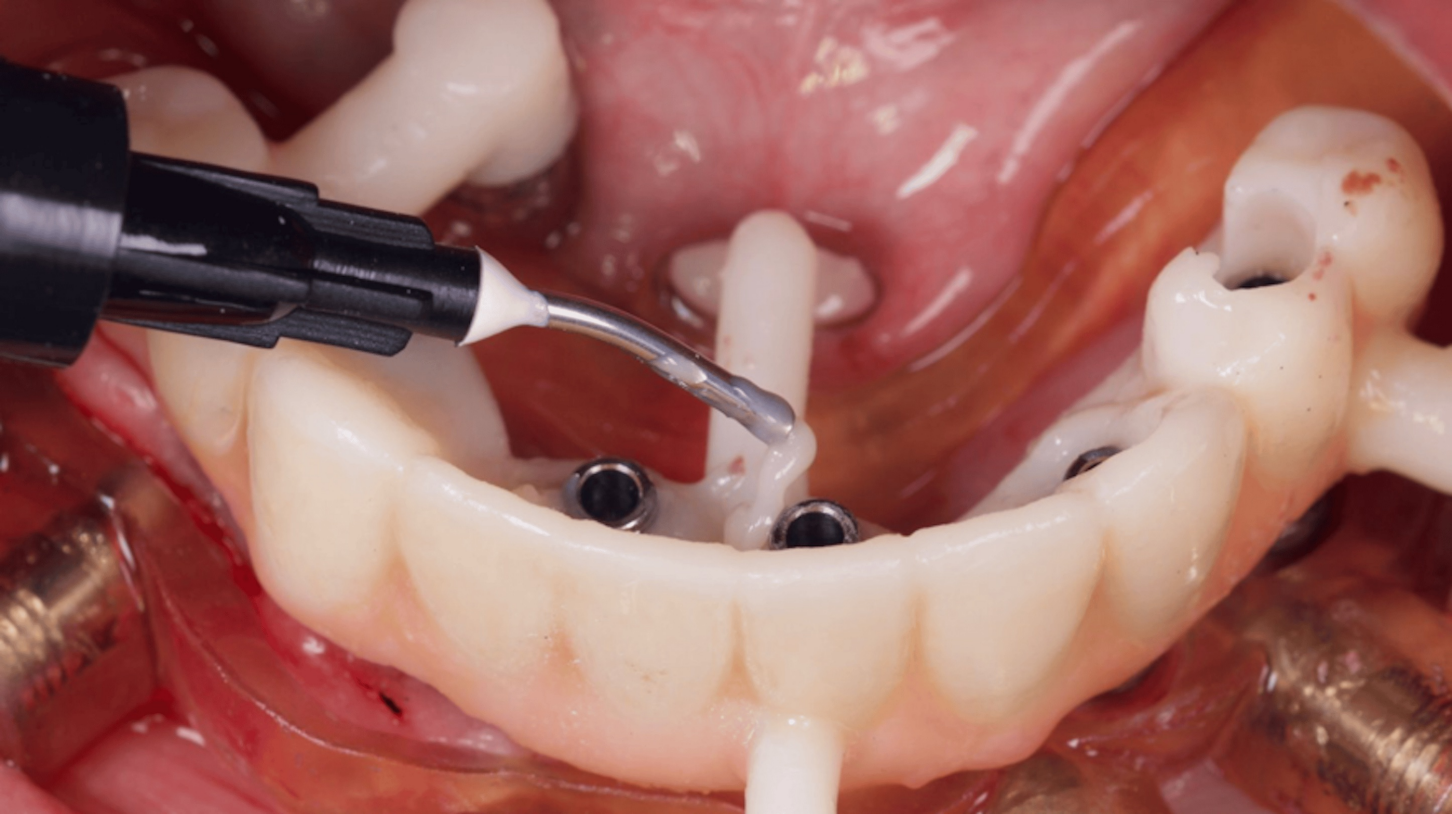
Capture of titanium cylinders to the temporary prosthesis with flow-resin

**Figure 14:**
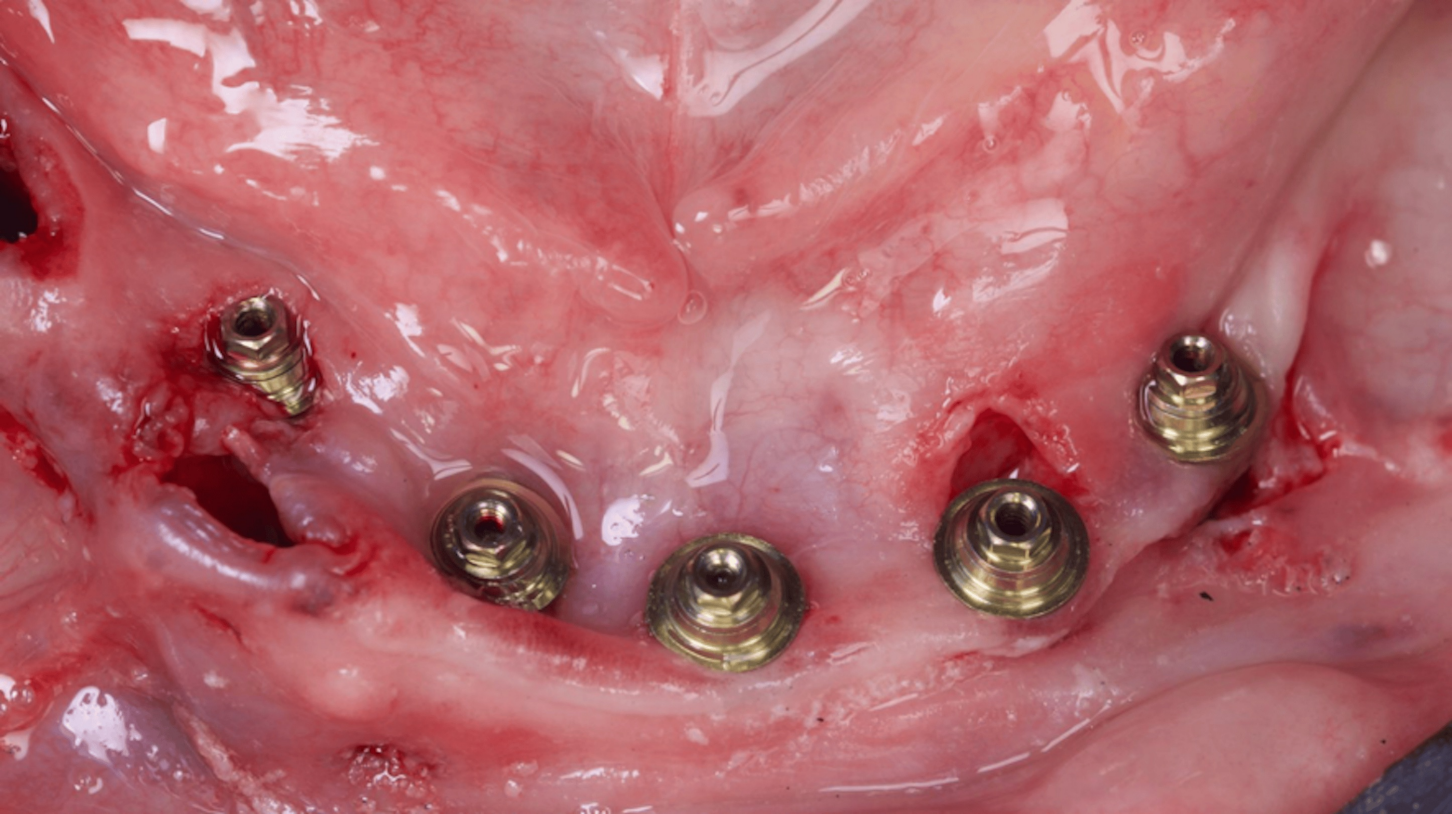
Oclusal view of the mucosa with the mini-abutments installed in the implants after removal of the surgical guides and temporary prosthesis

**Figure 15:**
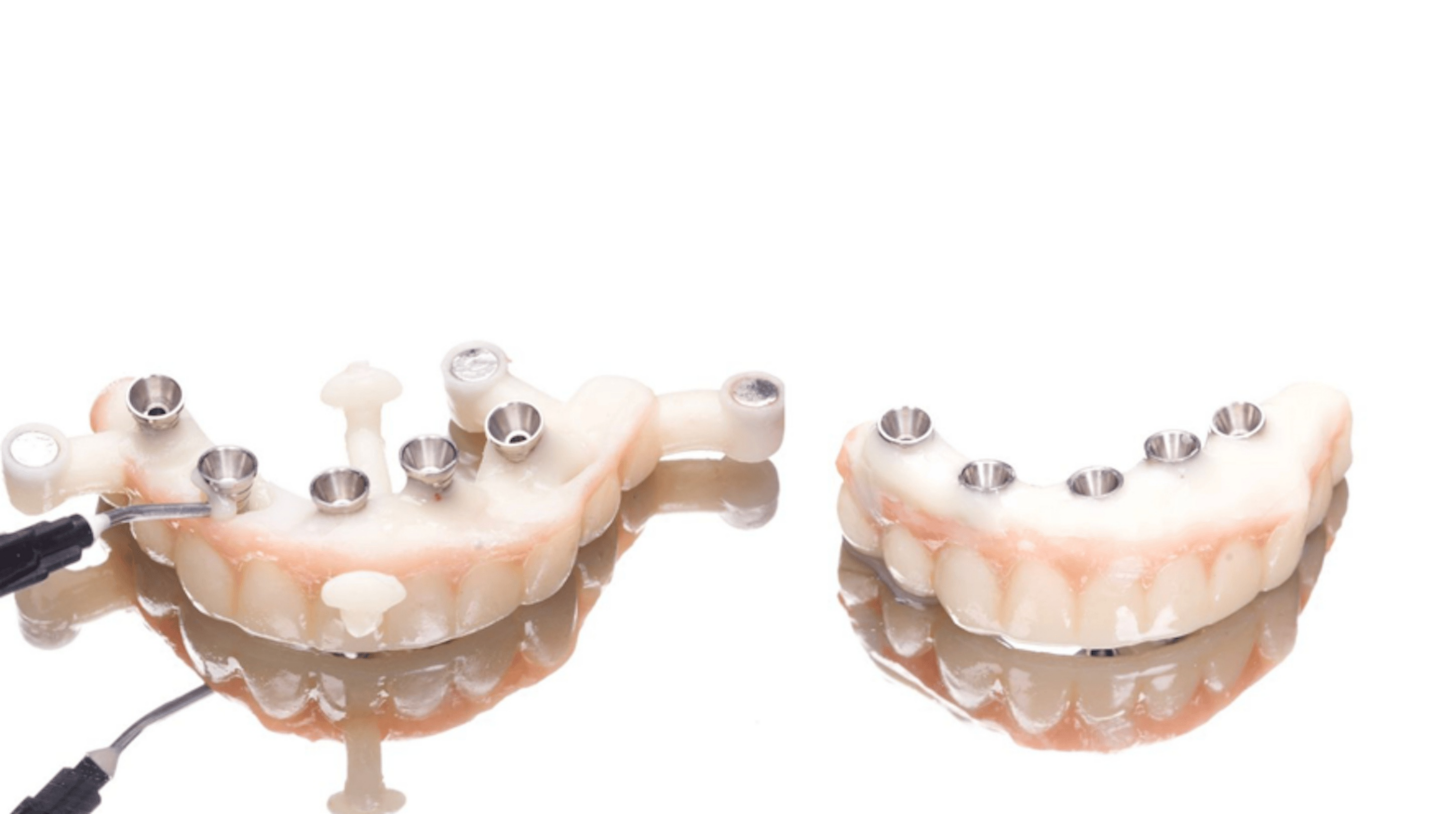
Filling of the gaps between the titanium cylinders and the temporary prosthesis with flowable resin (left) and stackable pins removal (right)

**Figure 16:**
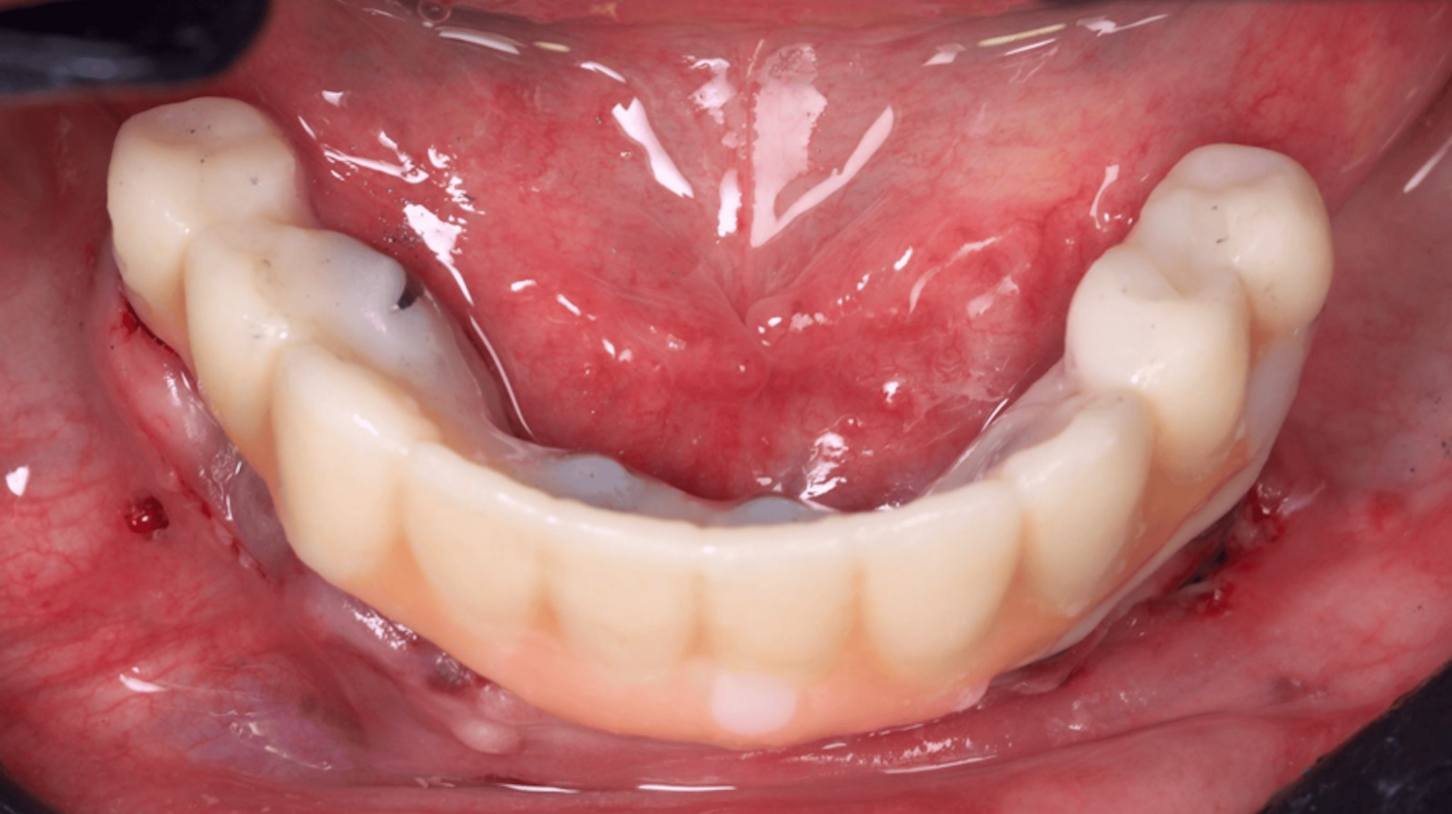
Oclusal view of the installed temporary fixed screw-retained prosthesis

**Figure 17:**
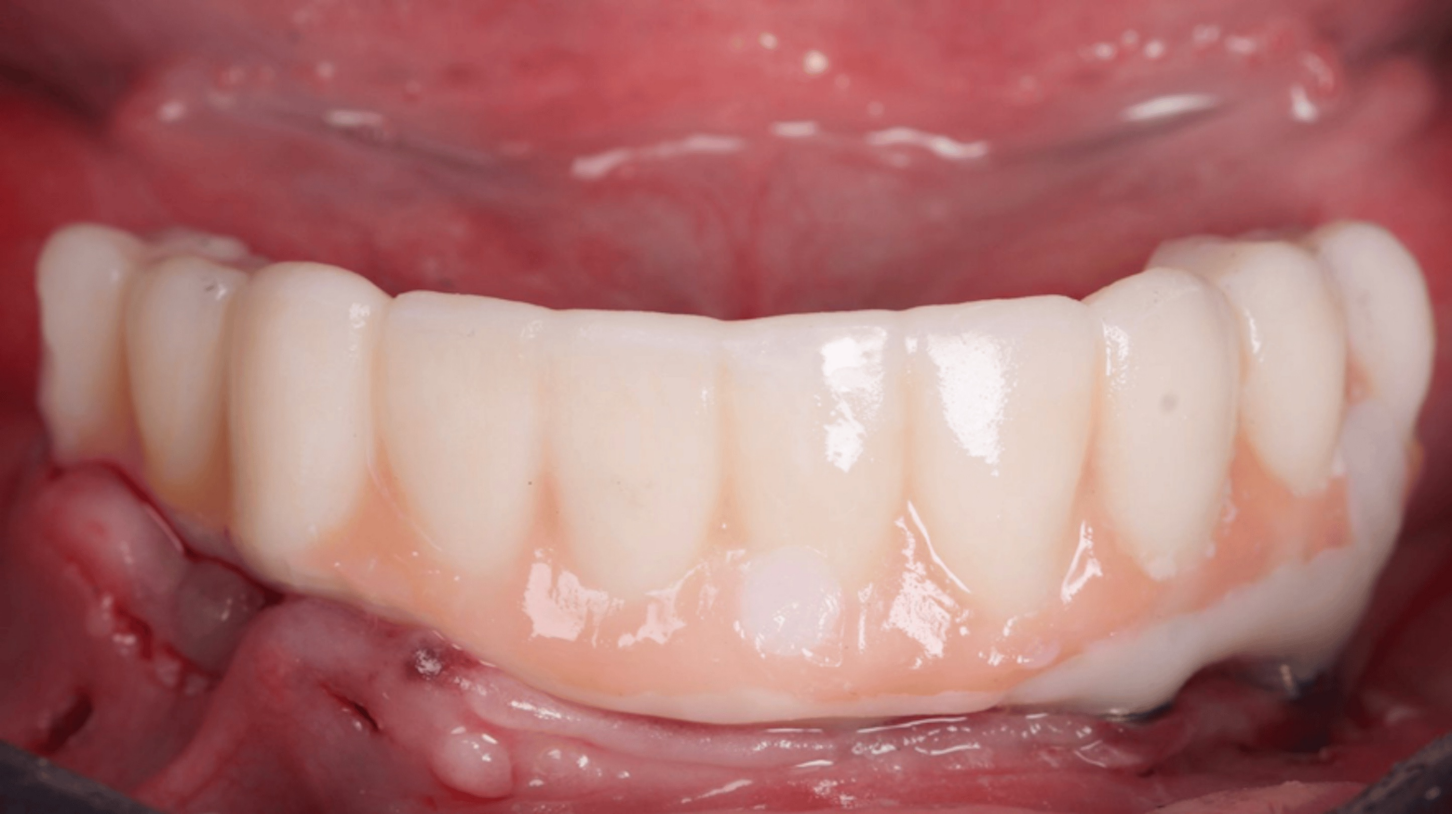
Buccal view of the installed temporary fixed screw-retained prosthesis

**Figure 18:**
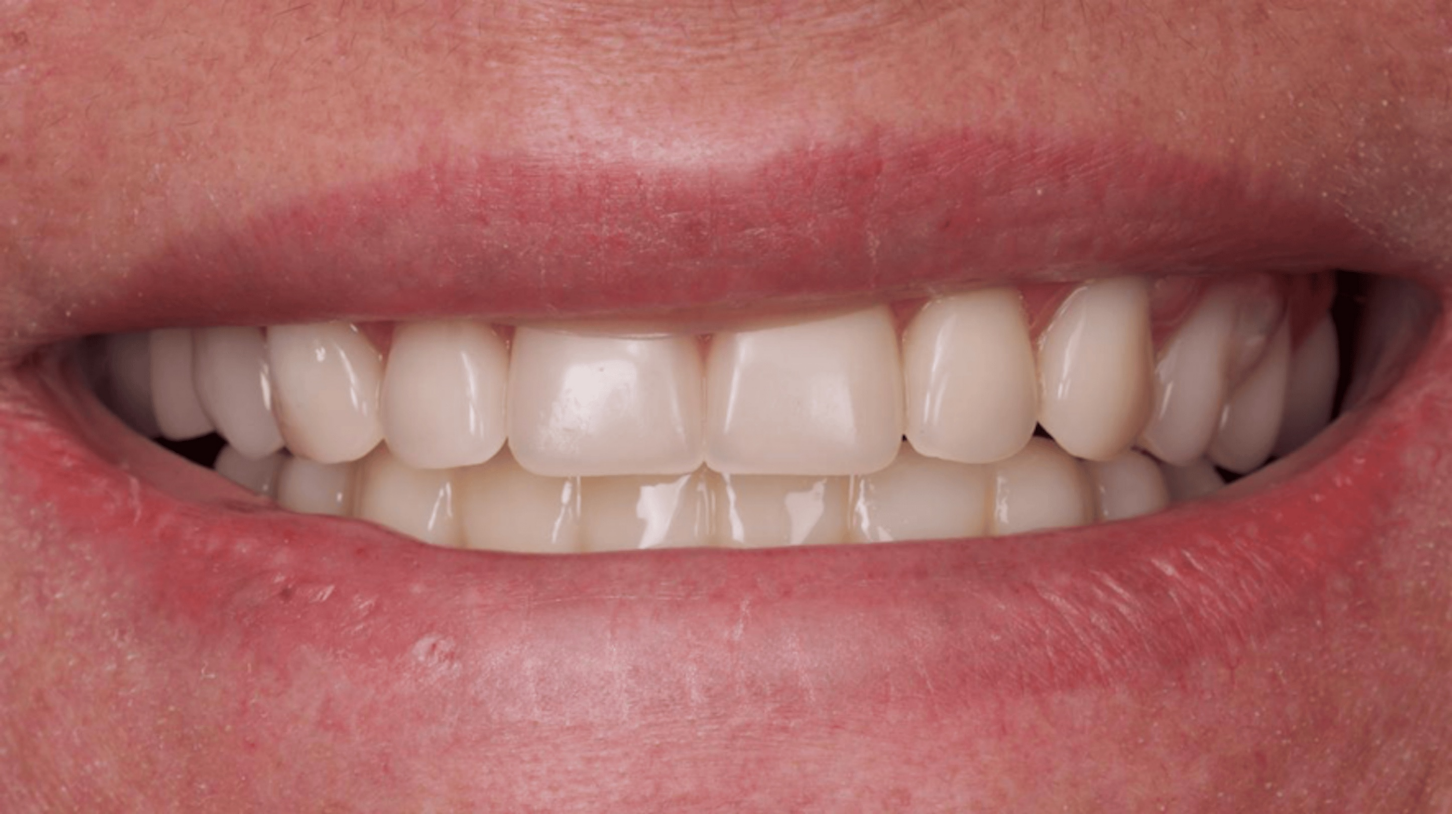
Extraoral view of the esthetics restored immediately after surgery

The 3D digital planning also allowed the crafting of a resin-printed (Makertech 3D Ltd., Birmingham, GBR) temporary fixed prosthesis (Figure 12), which was installed intraoperatively after the abutment installation in all implants and the use of flowable resin to fill the gaps between the provisional prosthesis and the titanium provisional cylinders (Figure 13), and before the removal of the surgical guide (Figure 14). After the proper resin polishing and adjustments (Figure 15), the fixed screw-retained temporary prosthesis was installed over the abutments (Figures 16-17), and the patient had aesthetics and function restored immediately after surgery (Figure 18). 

**Figure 19:**
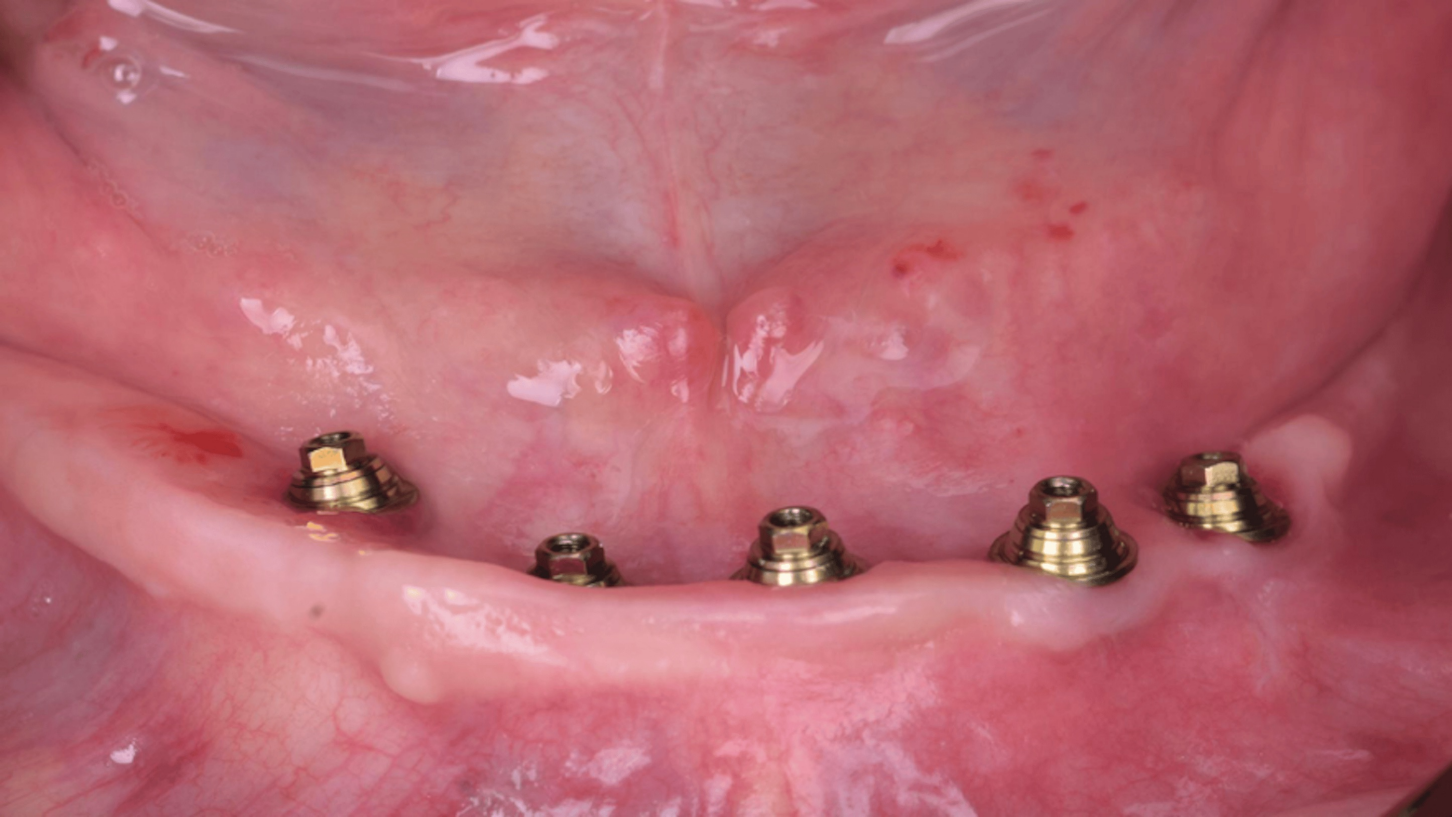
Buccal view of the mucosa and abutments after removal of the temporary prosthesis

**Figure 20:**
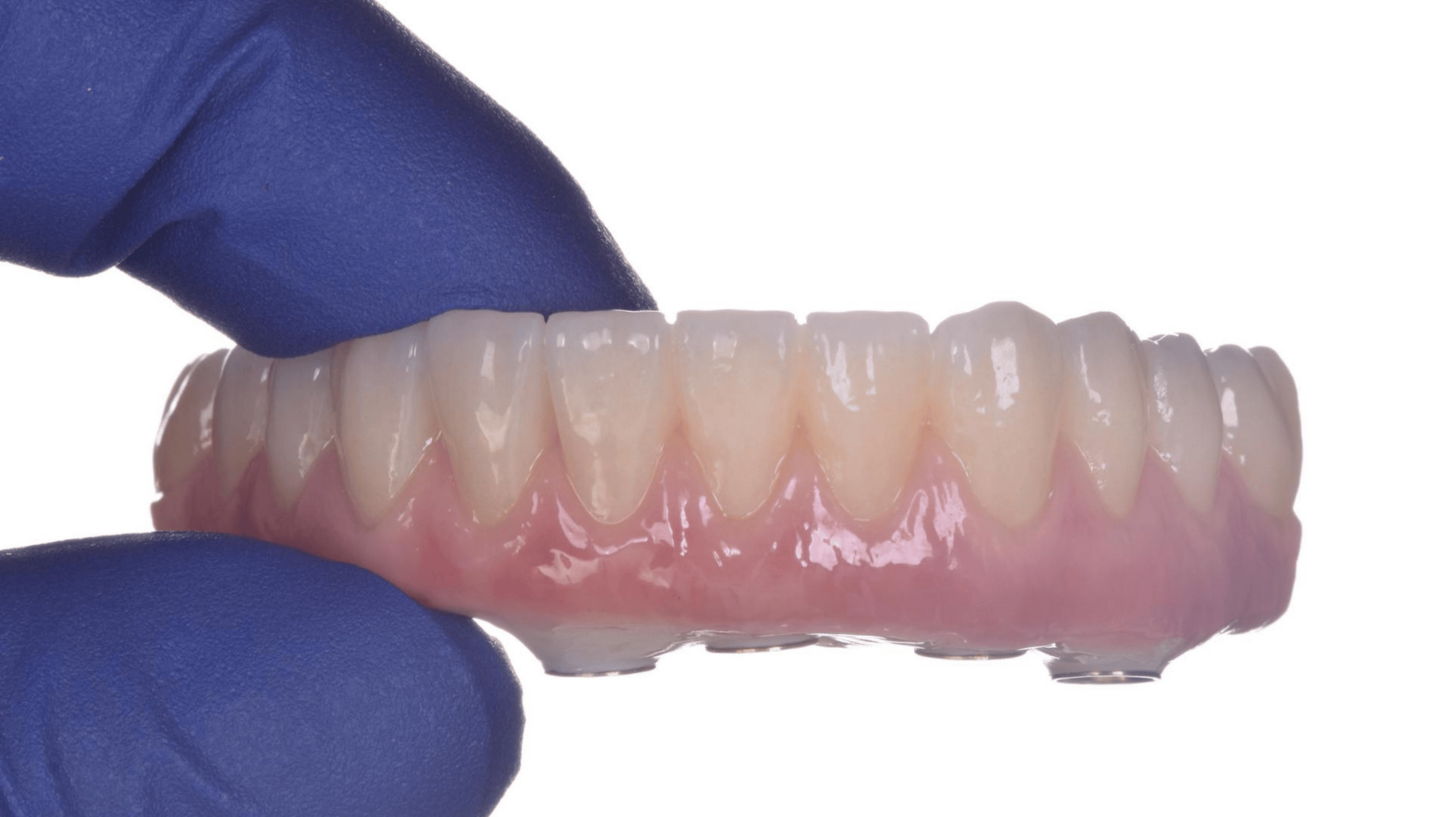
View of the zirconia rehabilitation before installation

**Figure 21:**
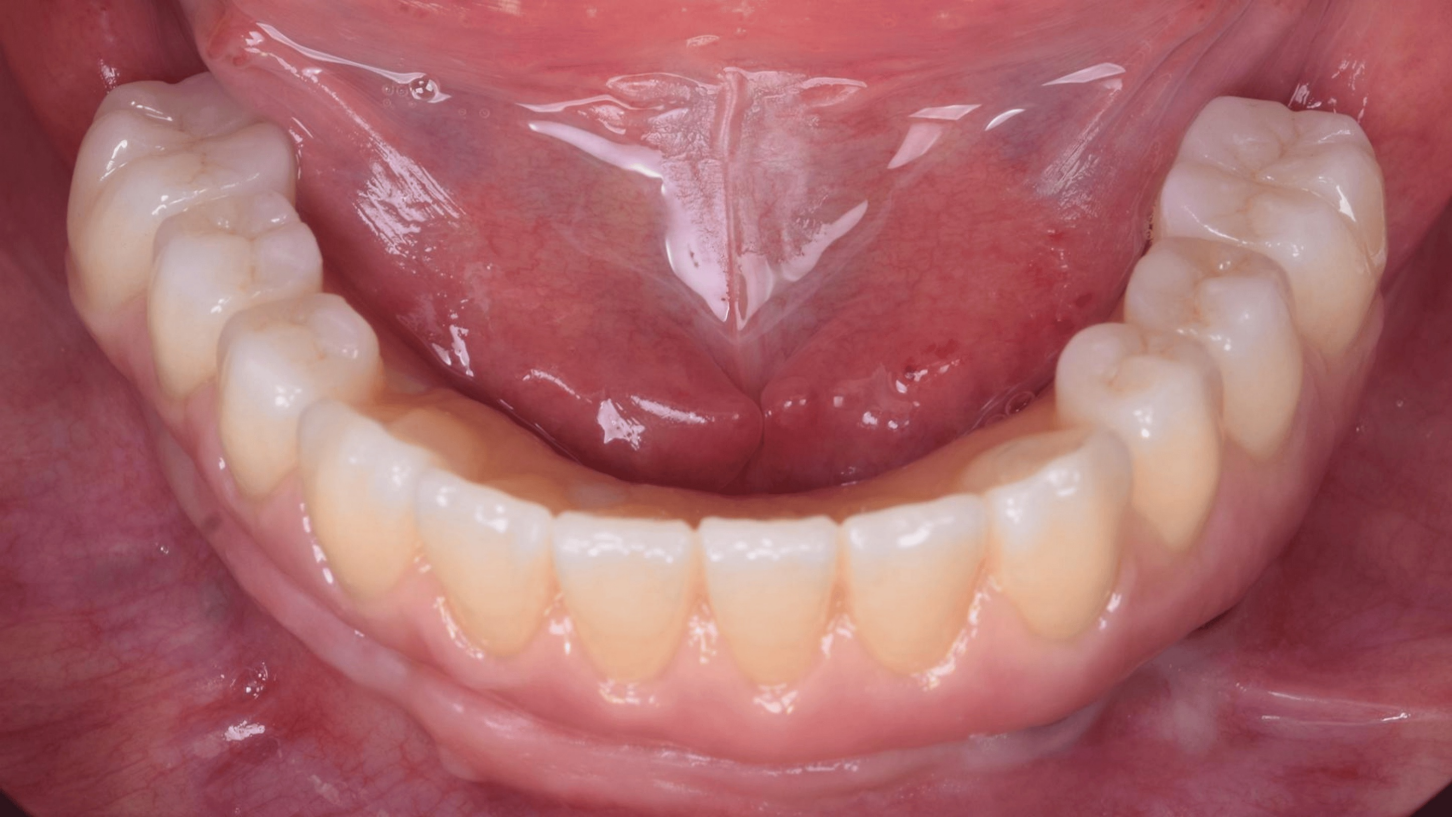
Buccal view of the installed zirconia-fixed rehabilitation

The patient was instructed to apply ice bags to the surgical area for 48 hours, for approximately 15 minutes every hour, and to eat only soft foods. On the day after surgery, the patient suspended the medication because she had no pain. After four months, the patient returned, and the temporary prosthesis was replaced for a final full-arch screw-retained zirconia rehabilitation (Figures 19-21).

**Figure 22:**
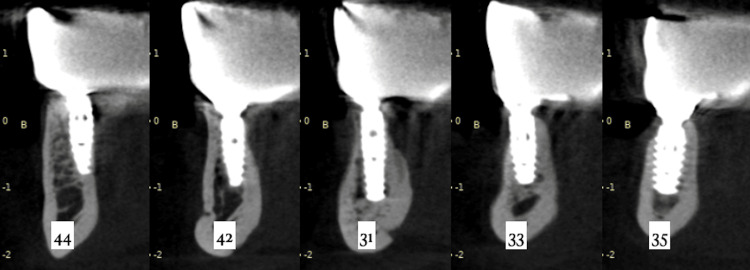
CBCT of the implants at the 24-month follow-up CBCT: Cone beam computed tomography

After 24 months, a cone beam computed tomography (CBCT) was performed for radiographic control of the implants and the surrounding bone (Figure 22), showing great stability of the peri-implant hard tissue. The peri-implant mucosa was noted to be healthy, without bleeding on probing. Peri-implant sulcus depth was lower than 3 mm, even around the left distal implant that didn’t have a satisfactory keratinized tissue band. This shows that the patient was able to maintain proper hygiene under the prosthesis. The patient reported great satisfaction with the oral rehabilitation, was very confident in public to smile, eat, and speak with higher self-esteem than before. 

## Discussion

Advances in 3D digital planning in implantology are not limited to planning implant positions, producing computed surgical guides, or preparing prosthetic rehabilitation even before surgery, with reliable accuracy, as presented in this case. Digital workflow in the 21st century has become a part of the dental offices and laboratories’ daily routine, such as intraoral digital scanning, virtual planning, guided surgery, and 3D printing [2,3,7,8]. Also, intraoral scanning has a vast advantage over conventional impression, considering it is more comfortable for patients, saves time and costs, besides avoiding mishaps like gypsum model fracture or loss [2,3,9,10]. In this case report, we could use most of the advantages of the latest digital workflow to deliver fixed rehabilitation over implants more predictably, faster, and with more comfort to the patient.

The surgical stackable guides warrant a faster and more precise surgical procedure, decreasing the trans-operative and post-operative morbidity since the surgeon can perform a flapless implant placement with a safer predictability of the distance between the implant and important anatomical structures such as the maxillary sinus and inferior alveolar nerve [10]. A flapless computed-guided surgery promotes greater comfort during and after the procedure. With such predictability, we could perform an immediate loading procedure with the installation of the temporary prosthesis intraoperatively, and immediately after placement of the implants and abutments [6,8]. As a consequence, the patient finishes the surgery with function and aesthetics restored [10].

Computer-guided, flapless implant surgery offers the advantages of high-precision implant placement, shorter surgical time, less trauma and bleeding, and less postoperative inflammation, edema, and hematoma. Among the disadvantages of flapless surgery, we can cite that it does not consider the soft tissue quality, and in certain situations, some of the implants may be placed in areas with little or no keratinized tissue [4,6,10]. In this case report, only one of the implants was placed in a region with minimum keratinized tissue, and needs more attention on the peri-implant health maintenance.

This case report described the placement of commercially available titanium dental implants produced by additive manufacturing (direct metal laser sintering). The layer-by-layer build-up of 3D printed implants offers versatility, accuracy, better mechanical properties, and enables better control of the implant micro and nano-geometry [11]. A recent study [12] showed a similar case report with a 24-month follow-up that used the same implant manufacturer, with soft and hard peri-implant tissue stability for this period. The same results are shown in our case report, with a different approach to the material in the prosthetic phases, both temporary and zirconia rehabilitation. Because in this case [12], the final prosthetic phase was all analogic, with the use of impressions; more appointments were necessary until finalization, which is another advantage of the full digital workflow. 

The combination of digital planning with the development of new technologies, such as dental implants produced by additive manufacturing, improved the bone response, facilitating wound healing in the early stages of osseointegration [13,14]. Previous studies in humans [13,14] and animals [15] showed that titanium implant surface topography produced by 3D printed technology provides increased bone volume and higher rates of bone-to-implant contact, resulting in stable bone anchorage. 

## Conclusions

This clinical case demonstrates how a fully digital workflow in implantology can significantly enhance the predictability, efficiency, and quality of full-arch rehabilitations. By integrating advanced technologies such as 3D imaging, virtual planning, surgical stackable guides, and additive manufacturing of implants and temporary prosthetics, clinicians can achieve precise implant positioning, minimize surgical trauma, and deliver immediate functional and esthetic outcomes.

The flapless approach, guided by stackable surgical templates, not only reduced patient discomfort and postoperative complications but also allowed for the immediate loading of a prefabricated temporary prosthesis. Furthermore, the use of 3D-printed titanium implants, with their enhanced surface properties, likely contributed to the favorable osseointegration and long-term stability observed at the 24-month follow-up.

This case highlights how digital dentistry is not merely an adjunct but a transformative tool that is reshaping the standards of care in implant-based oral rehabilitation. As these technologies continue to evolve, they promise even greater advancements in precision, patient comfort, and long-term success.
